# Comparison of a 36-week functional training program versus traditional physical education on physical fitness in male adolescents: a longitudinal school-based study

**DOI:** 10.3389/fpubh.2026.1936635

**Published:** 2026-09-08

**Authors:** Tao Li, Jianpeng Wu, Jinling Wei, Xiao Liu, Tianjie Yu

**Affiliations:** 1 College of Physical Education and Health, East China Normal University, Shanghai, China; 2 College of Physical Education, Guizhou University, Guiyang, China; 3 Shenzhen Qianhai Harbour Primary School of Shenzhen Qianhai Innovative Education Group, Shenzhen, China; 4 School of Computer and Computing Science, Hangzhou City University, Hangzhou, China

**Keywords:** adolescents, functional training, health promotion, pediatric health, physical education, physical fitness, school-based intervention

## Abstract

**Background:**

Evidence comparing functional training with traditional physical education in Chinese high schools is limited. This longitudinal study compared a 36-week functional training program versus standard physical education on fitness and health markers in male adolescents.

**Methods:**

Male adolescents (*N* = 670; aged 16–18 years) from 18 high schools were allocated to a functional training group (FTG, *n* = 351) or traditional physical education group (TPEG, *n* = 319). Nineteen fitness indicators were assessed at baseline (T1), post-Phase I at 18 weeks (T2), pre-Phase II after summer holiday (T3), and post-Phase II at 36 weeks (T4). Analyses compared T1 vs. T2 (18-week effects) and T1 vs. T4 (36-week effects); T3 served as a pre-Phase II reassessment and was not analyzed as an outcome time point. Linear mixed models (LMMs) with baseline adjustment and school-level random intercepts served as the primary analysis, with t-tests and repeated-measures ANOVA as secondary analyses.

**Results:**

LMMs revealed significant group effects favoring FTG for cardiovascular endurance (resting pulse: −2.34 bpm, 95% CI [−4.26, −0.42], d = 0.24; 1,000-m run: −15.36 s, 95% CI [−20.03, −10.69], d = 0.53), muscular strength (1RM squat: +2.81 kg, 95% CI [1.95, 3.67], d = 0.45; pull-ups: +0.61 reps, 95% CI [0.24, 0.98], d = 0.21), agility (T-test: −0.22 s, 95% CI [−0.40, −0.04], d = 0.26), and balance (closed-eye single-leg stance: +21.32 s, 95% CI [10.68, 31.96], d = 0.41). Time×group interactions were significant for BMI (*p* = 0.012), body fat percentage (*p* = 0.003), lung capacity (*p* < 0.001), 1RM squat (*p* < 0.001), pull-ups (*p* = 0.001), and maximum squat repetitions (*p* = 0.006). Movement quality indicators (FMS) showed significant effects only after 36 weeks, with FTG attenuating the decline in trunk stability (*p* = 0.026), hurdle steps (*p* = 0.012), and rotational stability (*p* = 0.040) compared to TPEG. Body composition showed limited between-group differences at individual time points despite significant interactions.

**Conclusion:**

A 36-week structured progressive exercise program was associated with greater improvements in cardiovascular endurance, muscular strength, and agility than traditional physical education among male adolescents, with duration-dependent adaptations. However, differential attrition (FTG: 46.7% vs. TPEG: 37.3% at 36 weeks) and the absence of dietary control warrant cautious interpretation.

## 1 Introduction

Adolescence represents a critical developmental window during which high school students experience accelerated physiological maturation and establish health-related behaviors that track into adulthood (1). The high school period (ages 15–18) is a pivotal phase for enhancing physical fitness and developing movement competencies that underpin lifelong participation in physical activity and long-term health (2). Timely, scientifically designed interventions during this stage can solidify fitness foundations, promote physiological adaptation, and prepare students for healthy adult lives (3).

The significance of adolescent physical fitness extends beyond athletic performance. Epidemiological evidence establishes robust associations between cardiorespiratory fitness, muscular strength, and metabolic health, with profound implications for reducing cardiovascular disease risk (3). The American Heart Association has designated cardiorespiratory fitness as a clinical vital sign, recognizing its predictive validity for mortality and cardiovascular events (4). For high school students navigating academic demands and physical development, cultivating comprehensive fitness encompassing both health-related and skill-related components represents a public health imperative (5).

Contemporary research demonstrates that structured physical activity interventions yield multifaceted health benefits. Significant improvements in muscular fitness, movement competency, and psychological outcomes have been reported following resistance training integrated into physical education curricula (6). Physical education must prioritize meaningful fitness outcomes that enhance self-efficacy and sustained participation in physical activity (7). Such interventions are also associated with improvements in BMI, respiratory function, muscular fitness, and cognitive performance (8, 9).

Despite this evidence, a gap persists between exercise science and secondary education practice. Traditional curricula emphasize sport-specific skills over foundational movement capacities (10, 11), and functional training, emphasizing movement quality, core stability, and neuromuscular control, remains poorly integrated (12). Functional training offers a compelling framework for adolescent health, emphasizing transfer to real-world activities (13) and aligning with adolescents’ needs for enhanced balance, coordination, and agility, thereby reducing injury risk (12, 14). Recent studies have demonstrated the effectiveness of functional training in improving fundamental motor skills, muscular fitness, and movement quality in adolescent populations (15, 16). School-based functional training programs have been shown to enhance physical fitness outcomes compared to traditional physical education, with benefits observed in muscular strength, coordination, and balance (17, 18). However, evidence from Chinese high school settings remains limited, particularly regarding long-term (≥36 weeks) interventions that assess both health-related and skill-related fitness outcomes.

The concept of movement competency, encompassing flexibility, agility, coordination, balance, and stability, has emerged as critical for adolescent health (19). Balance reflects neuromuscular control efficiency (20), while coordination training has been shown to improve physical fitness capabilities in adolescent populations (21). Body composition represents another critical health dimension, with optimal fat-to-lean mass supporting metabolic function (22). Physical activity interventions show variable effectiveness in modifying composition, with optimal health outcomes within specific BMI ranges (23, 24).

Despite this rationale, significant research gaps persist: (1) limited examination of intervention duration effects across an academic year; (2) inadequate characterization of differential responsiveness among health-related versus skill-related fitness components; (3) insufficient validation of functional training within general high school curricula; and (4) limited investigation of interactions between natural development and structured training (25, 26). Although recent studies have demonstrated the effectiveness of functional training in improving fundamental motor skills, muscular fitness, and movement quality in adolescent populations (15, 16), evidence from Chinese high school settings remains limited, particularly regarding long-term (≥36 weeks) interventions that assess both health-related and skill-related fitness outcomes.

The present study addresses these gaps through a longitudinal investigation conducted in authentic high school settings. Over 36 weeks, across 18 schools, 670 male adolescents participated in either a structured functional training program or continued with standard physical education, with 19 fitness indicators spanning 11 categories assessed at baseline, 18 weeks, and 36 weeks. This design enabled evaluation of both the intervention’s effects and the time course of adaptations. Three hypotheses guided the analysis: first, that functional training would be associated with greater fitness improvements than traditional physical education; second, that improvements would show duration-dependent effects, being more pronounced at 36 weeks than at 18 weeks; and third, that fitness categories would exhibit differential responsiveness, with cardiovascular endurance and muscular strength showing more consistent effects than body composition measures.

## 2 Materials and methods

### 2.1 Research participants

This study recruited 670 male students (aged 16–18 years) from 18 high schools across the provinces of Guizhou, Shandong, and Sichuan. Inclusion criteria were: (1) uncorrected visual acuity ≥4.8; (2) height 163–185 cm; and (3) National Student Physical Fitness Test (NSFHS) score ≥60. The visual acuity criterion ensured adequate sensory input for balance and coordination tasks; the height range corresponded to the 5th-95th percentiles for Chinese male adolescents of this age; and the NSFHS threshold was selected to minimize injury risk during 1RM testing, as adolescents with clinically low fitness may have reduced neuromuscular control. These criteria were established to ensure participant safety and to create a relatively homogeneous sample for this proof-of-concept efficacy study. Of the 892 students initially assessed, 222 (24.9%) were excluded: 78 (8.7%) for visual acuity <4.8, 52 (5.8%) for height outside 163–185 cm, and 92 (10.3%) for NSFHS score <60. These restrictions necessarily limit generalizability to the broader adolescent population; the findings should be interpreted as proof-of-concept for a specific subgroup and should not be generalized to female adolescents, shorter/taller adolescents, or those with lower baseline fitness.

*Recruitment and school selection*: Schools were selected based on administrative convenience across three provinces (Guizhou, Shandong, and Sichuan). In some schools (e.g., Suiyang No. 1, Tongzi No. 1), only five students met the criteria due to small male enrollment or low NSFHS scores. All eligible students were recruited. School-level distribution is presented in Supplementary Table S1.

*Group allocation:* Within each participating school, eligible students were individually allocated to either the functional training group (FTG) or traditional physical education group (TPEG) using a computer-generated random number sequence (SPSS v26.0) with allocation concealment via sequentially numbered opaque sealed envelopes. Individual-level allocation was feasible because each school had sufficient student numbers to accommodate both conditions. To minimize contamination, FTG and TPEG students attended separate physical education sessions at different times, and instructors were trained and monitored to deliver only their assigned curriculum. No matching or stratification was applied. This was not a cluster-randomized trial; allocation occurred at the individual level within each school. Table 1 presents baseline characteristics demonstrating group comparability.

**Table 1:** Baseline characteristics and distribution of study participants.

Characteristic	Functional training group (*n* = 351)	Traditional PE group (n = 319)	Total (*N* = 670)	Between-group comparison
Age (years), mean ± SD	17.2 ± 0.8	17.1 ± 0.9	17.2 ± 0.8	t (668) = 1.52, *p* = 0.129
Height (cm), mean ± SD	174.3 ± 5.2	173.9 ± 5.4	174.1 ± 5.3	t (668) = 1.26, *p* = 0.208
Weight (kg), mean ± SD	68.5 ± 8.7	67.9 ± 9.1	68.2 ± 8.9	t (668) = 1.08, *p* = 0.281
NSFHS baseline score, mean ± SD	76.4 ± 8.2	75.8 ± 8.5	76.1 ± 8.3	t (668) = 1.33, *p* = 0.184
Number of participating schools	18	18	18	—
Geographic distribution	Guizhou, Shandong, Sichuan	Guizhou, Shandong, Sichuan	Guizhou, Shandong, Sichuan	—

NSFHS = National Student Fitness Health Standards. No significant differences were observed between groups at baseline (*p* > 0.05 for all comparisons). Between-group comparisons used independent t-tests; primary analyses utilized Linear Mixed Models with school-level random intercepts to account for the nested structure of students within schools.

### 2.2 Research methods

#### 2.2.1 Literature review

A comprehensive literature review was conducted using CNKI, Web of Science, and ProQuest databases. The search strategy employed the following keywords and Boolean combinations: (“high school students” OR “adolescents” OR “youth”) AND (“physical fitness” OR “physical activity”) AND (“functional training” OR “functional exercise” OR “resistance training”) AND (“intervention” OR “program”). The search was limited to peer-reviewed articles published in English or Chinese between 2000 and 2025. Reference lists of included articles were also manually screened to identify additional relevant studies. This review synthesized current evidence on training methodologies and intervention design, providing the theoretical foundation for the experimental protocol.

#### 2.2.2 Experimental design

*Variables:* The independent variables comprised group (FTG versus TPEG) and time (two academic semesters, totaling 36 weeks).

*Control measures:* To minimize the influence of extraneous variables, several control measures were implemented. All physical education instructors had comparable teaching experience and received centralized training on intervention protocols to ensure consistent delivery. Class hours and extracurricular schedules were standardized across both groups. Throughout the intervention period, potential confounding factors, including weather conditions, facility availability, and participant emotional states, were systematically documented and monitored. No matching or stratification procedures were applied at baseline. The dependent variables included 19 physical fitness indicators spanning 11 categories, as detailed in Table 2.

**Table 2:** Classification of dependent variables by fitness component.

Fitness category	Specific indicators	Measurement unit
Body composition	Body mass index (BMI)	kg/m^2^
	Wilk’s index	dimensionless
	Body fat percentage	%
	Waist-to-hip ratio	ratio
Cardiovascular endurance	Resting pulse	bpm
	Lung capacity	mL
	1,000-metre run	seconds
Muscular strength	1RM squat	kg
	Pull-ups	repetitions
	FMS trunk stability push-up	score (0–3)
Muscular endurance	Eight-level abdominal bridge	level
	Maximum bodyweight squat repetitions	repetitions
Flexibility	Sit-and-reach test	cm
Displacement speed	50-metre run	seconds
Coordination	FMS hurdle step	score (0–3)
Agility	T-test	seconds
Explosive power	Standing long jump	cm
Balance	Closed-eye single-leg stance	seconds
	FMS rotational stability test	score (0–3)

FMS, Functional Movement Screen; 1RM, one-repetition maximum; bpm, beats per minute; BMI, body mass index.

*Intervention protocol:* The intervention used a progressive two-phase structure: Phase I (foundational motor function: stability, flexibility, motor control) and Phase II (core stability, lower-limb conditioning). The weekly schedule comprised two 45-min in-class sessions and two 60-min extracurricular sessions, totaling approximately 210 min per week. Each 45-min session included warm-up (10 min), main component (30 min), and cool-down (5 min). Each 60-min extracurricular session included sport-specific drills (jumping, landing mechanics, change of direction) and small-sided games. The main component consisted of circuit-style exercises including squats (3 sets × 12–15 reps), lunges (3 × 10–12 per leg), planks (3 × 30–60 s), push-ups (3 × 10–15), balance drills (3 × 30 s per leg), and agility ladder drills (3 × 30 s). Rest intervals were 60–90 s between sets. External loads were bodyweight for Phase I; weighted vests (2–5 kg) and dumbbells (2–8 kg) were introduced in Phase II based on individual progression criteria.

*Training progression:* Intensity was monitored using RPE (target: 12–15 on Borg 6–20 scale), with progression every 4–6 weeks when ≥80% of participants met criteria: (1) load/volume increased 5–10% with good form; (2) exercise difficulty progressed (e.g., bilateral to unilateral, stable to unstable). Phase-specific progression was as follows: Phase I (weeks 1–18) focused on foundational motor function with bodyweight exercises and high repetitions (12–15 reps); Phase II (weeks 19–36) advanced to core stability and lower-limb conditioning with added external loads (weighted vests 2–5 kg; dumbbells 2–8 kg) and reduced repetitions (8–12 reps). Modifications for individual participants were permitted when RPE exceeded 15 or form was compromised, with exercises regressed to an easier variation (e.g., knee push-ups, supported lunges).

*Control group activities:* The traditional physical education group received an identical time allocation for standard physical education (basketball, volleyball, football) and extracurricular activities (basketball, calisthenics, running, badminton, shuttlecock), delivered without structured progression or functional emphasis.

*Fidelity monitoring:* All instructors (*n* = 36) received an 8-h workshop and manual; research staff conducted unannounced site visits (two per school per phase) with fidelity >90%. Fidelity was calculated as the percentage of session components completed as prescribed (warm-up, main component, cool-down; correct exercise technique; appropriate intensity monitoring). Attendance was recorded (adherence ≥75%). To minimize contamination between FTG and TPEG students within the same schools, FTG and TPEG students attended separate physical education sessions at different times, and instructors were trained and monitored to deliver only their assigned curriculum.

*Experimental procedure:* The experiment was conducted in two stages: March–July 2021 (Stage 1) and September 2021–January 2022 (Stage 2), spanning 36 weeks, including a two-month summer holiday between phases. One week before commencement, all participants completed baseline assessments. During the holiday, no structured monitoring was implemented; participants were not required to maintain any exercise regimen, and both groups were equally affected. Following baseline testing, the FTG commenced the structured program while the TPEG continued standard activities, with both groups maintaining identical contact hours. Post-testing was conducted immediately following each stage using identical protocols, with additional communication emphasizing consistent effort during assessments to minimize testing bias.

#### 2.2.3 Testing procedures

All testing procedures were standardized through preliminary training sessions for physical education teachers. These sessions addressed both technical execution (“how to obtain accurate data”) and conceptual understanding (“why these measurements are taken”). Instructional videos were distributed to all participating schools, and research staff conducted on-site supervision during testing periods. Standardized equipment guidelines were provided to minimize systematic measurement error across schools. Seven of the 19 fitness indicators were derived from the (NSFHS). Testing protocols for all measures followed established guidelines to ensure reliability and validity. Assessors were blinded to group allocation; the same assessors evaluated participants at all time points within each school. Inter-rater reliability was established (ICC > 0.90 for all measures). Test–retest reliability was determined using a subsample of 30 participants assessed one week apart (ICC = 0.85–0.95 across measures). Equipment specifications included calibrated digital scales (SECA 813), stadiometer (SECA 213), and standard gym equipment (Eleiko); calibration was performed weekly. For 1RM squat testing, safety procedures included familiarization, spotting, standardized warm-up, and progressive loading (5–10 kg increments).

#### 2.2.4 Statistical analysis

*Data collection:* Data were collected at four time points: March 2021 (baseline, T1), July 2021 (post-Phase I, 18 weeks, T2), September 2021 (pre-Phase II, after summer holiday, T3), and January 2022 (post-Phase II, 36 weeks, T4). Analyses compared T1 vs. T2 (18-week effects) and T1 vs. T4 (36-week effects); T3 served as a pre-Phase II reassessment to monitor changes during the summer holiday but was not analyzed as a primary outcome time point. School vice principals, political education directors, and homeroom teachers coordinated data collection, while physical education teachers conducted fitness assessments.

*Data processing:* Raw data were cleaned and organized to ensure integrity before analysis. Descriptive statistics were calculated for all fitness indicators at each time point using SPSS version 26.0.

*Primary analysis - linear mixed models (LMMs):* LMMs were employed as the primary analytic approach, including all 670 allocated participants (intention-to-treat; all participants were analyzed according to their original group allocation, regardless of adherence or dropout). LMMs use all available data at each time point without imputation (i.e., no listwise deletion), providing unbiased estimates under the Missing At Random assumption. Multiple imputation sensitivity analyses (20 imputations) were conducted to assess the robustness of findings to different missing-data assumptions (Supplementary Table S6). LMMs offer several advantages: (a) baseline values included as covariates to adjust for initial between-group differences; (b) all available data used without excluding participants with missing follow-up; (c) random intercepts for participants account for within-participant correlation; (d) random intercepts for schools were included to account for the nested structure of students within schools; and (e) adjustment for covariates including age and baseline fitness score. Models included fixed effects for time (categorical: T1, T2, T4), group (FTG vs. TPEG), and the time×group interaction, with random intercepts for participants and schools. The time×group interaction was the primary effect of interest, representing differential changes between groups over time. Restricted maximum likelihood (REML) estimation was used with an unstructured covariance structure. Baseline values were included as covariates to adjust for initial between-group differences. Model assumptions were verified by inspecting residual plots and Q-Q plots. Degrees of freedom were estimated using the Satterthwaite approximation. Variance components and ICCs were estimated to quantify school-level clustering effects and are presented in Supplementary Table S2. The analytical code for all statistical models is available from the corresponding author upon reasonable request.

*Secondary analyses:* Independent-samples t-tests (between-group comparisons of change scores) and paired-samples t-tests (within-group comparisons) were conducted as secondary analyses to facilitate comparison with prior studies. Repeated-measures ANOVA with Greenhouse–Geisser correction was used to examine time×group interactions across the three primary outcome time points (T1, T2, and T4). For ordinal outcomes (FMS scores), Mann–Whitney U tests were used for between-group comparisons.

*Effect sizes:* Standardized effect sizes were calculated using Cohen’s d for t-tests (with pooled standard deviations) and partial eta squared (η^2^p) for ANOVA. For Cohen’s d, values of 0.20, 0.50, and 0.80 were interpreted as small, moderate, and large effects, respectively.

*Multiple comparisons*: To address the risk of Type I error from testing 19 outcomes, the False Discovery Rate (FDR) correction (Benjamini-Hochberg procedure) was applied to all primary and secondary analyses. Both unadjusted and FDR-adjusted *p*-values are reported for transparency. Primary outcomes were prespecified as cardiovascular endurance (1000-m run) and muscular strength (1RM squat), as these were hypothesized to show the most consistent effects. All other fitness indicators were considered secondary outcome.

*Sample size calculation:* Assuming a moderate effect size (Cohen’s d = 0.30) for the primary outcomes (1000-m run and 1RM squat), with 80% power, α = 0.05, and an intraclass correlation coefficient (ICC) of 0.10 to account for school-level clustering, a minimum of 480 participants was required. Our final sample of 670 participants provided sufficient power to detect the anticipated effects.

*Missing data and attrition analysis:* To assess potential bias from differential attrition, baseline characteristics were compared between completers and dropouts within each group using independent t-tests and chi-square tests. LMMs are robust to missing data under the Missing At Random (MAR) assumption; this assumption was evaluated by comparing baseline characteristics between completers and dropouts. Although LMMs provide unbiased estimates under the MAR assumption, we acknowledge that dropout was significantly associated with FTG allocation (OR = 1.62, *p* = 0.006), raising the possibility of informative missingness. While the similarity of baseline characteristics between completers and dropouts (Table 3) and the consistency of results with multiple imputation sensitivity analyses (Supplementary Table S6) support the MAR assumption, we cannot fully exclude the possibility that unmeasured factors influenced dropout. If missingness were informative, the estimated intervention effects may be overestimated, as more motivated or fitter participants may have been more likely to remain in the FTG. Therefore, the findings should be interpreted with appropriate caution. To formally test duration-dependent effects (H2), a prespecified contrast was conducted comparing the intervention effect at 18 weeks with the intervention effect at 36 weeks within the LMM framework. This contrast directly tested whether the FTG-TPEG difference was significantly larger at 36 weeks than at 18 weeks. Statistical significance was set at α = 0.05 for primary analyses. All reported *p*-values are two-tailed.

**Table 3:** Attrition analysis: dropout characteristics, missing data pattern, and withdrawal reasons.

Characteristic	FTG		TPEG		*p*-value
	Completers (*n* = 187)	Dropouts (*n* = 164)	Completers (*n* = 200)	Dropouts (*n* = 119)	
Baseline characteristics (Completers vs. Dropouts)					
Age (years)	17.1 ± 0.8	17.3 ± 0.9	17.0 ± 0.8	17.2 ± 0.9	0.184/0.205
BMI (kg/m^2^)	22.4 ± 3.1	22.6 ± 3.3	22.3 ± 3.0	22.5 ± 3.2	0.561/0.572
NSFHS score	76.8 ± 8.1	75.9 ± 8.3	76.2 ± 8.4	75.3 ± 8.7	0.301/0.342
1,000-m run (s)	246.3 ± 28.5	248.1 ± 29.2	247.5 ± 28.9	249.2 ± 30.1	0.561/0.601

Missing data pattern	FTG (*n* = 351)	TPEG (*n* = 319)
Phase I (18 weeks)		
Completed	293 (83.5%)	293 (91.8%)
Dropped out	58 (16.5%)	26 (8.2%)
Phase II (36 weeks)		
Completed	187 (53.3%)	200 (62.7%)
Dropped out	164 (46.7%)	119 (37.3%)

Reasons for withdrawal	FTG Phase I	FTG Phase II	TPEG Phase I	TPEG Phase II
Physical reasons				
Injury (unrelated to intervention)	8	10	5	9
Illness	10	16	6	12
Family relocation	6	12	4	8
Psychological reasons				
Lack of motivation	16	34	5	32
Time constraints	12	26	4	24
Other	6	8	2	8
Total	58	106	26	93

No significant differences were observed between completers and dropouts within either group for baseline characteristics (*p* > 0.05 for all comparisons). Phase II dropout totals represent cumulative attrition; new dropouts during Phase II were FTG = 106 and TPEG = 93. No withdrawals were attributed to injuries from the intervention.

#### 2.2.5 Ethical considerations

This study received ethical approval from the Human Medical Experiment Ethics Committee of Guizhou University (Approval No. HMEE-GZU-T078). The committee explicitly approved the use of passive parental consent and verbal student assent, as the study involved comparison of two standard educational approaches with established safety profiles and the risk to participants was not greater than that associated with routine physical education participation. A school-mediated verbal consent-and-assent procedure was therefore implemented. Parents/guardians were informed through parent-teacher meetings and provided with written information sheets, with passive (opt-out) consent employed (parents were given two weeks to object to participation; the absence of an objection was formally recorded as consent). Student verbal assent was obtained at baseline and reaffirmed before each data collection or training session. The ethics committee’s approval letter (HMEE-GZU-T078) explicitly documents this consent procedure. Participant safeguards included a guaranteed right to withdraw at any time, immediate data anonymization, and supervision of all activities by qualified personnel.

## 3 Results

### 3.1 Participant flow and attrition

**Figure 1:**
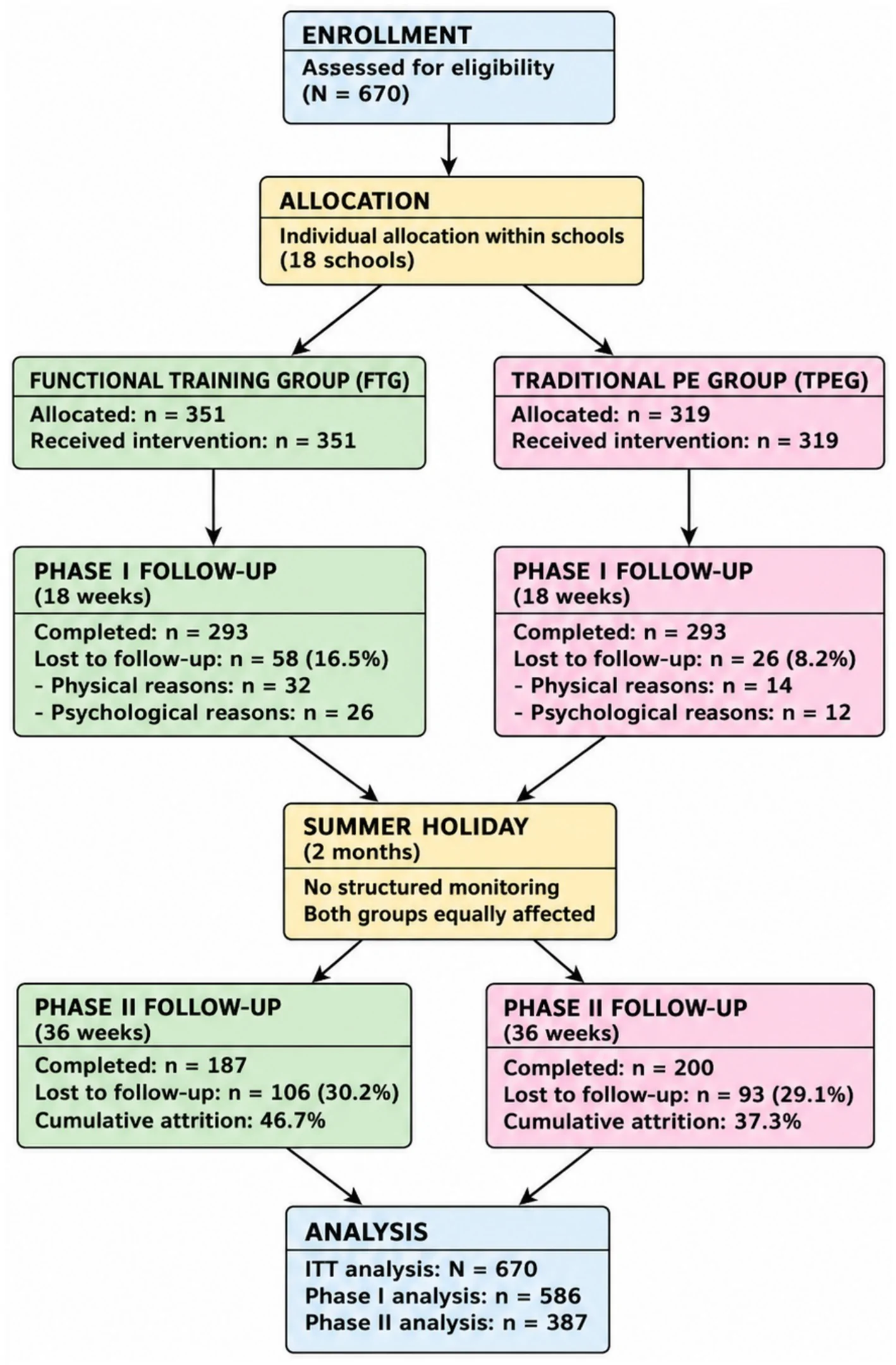
Participant flow diagram. Enrollment, individual allocation within schools, follow-up, and analysis of 670 male high school students across the 36-week intervention period. Phase I (18-week) analysis: FTG *n* = 293; TPEG *n* = 293. Phase II (36-week) analysis: FTG *n* = 187; TPEG *n* = 200. Lost to follow-up includes withdrawals due to physical or psychological reasons. Allocation was performed at the individual level within each participating school (not school-level randomization).

**Figure 2:**
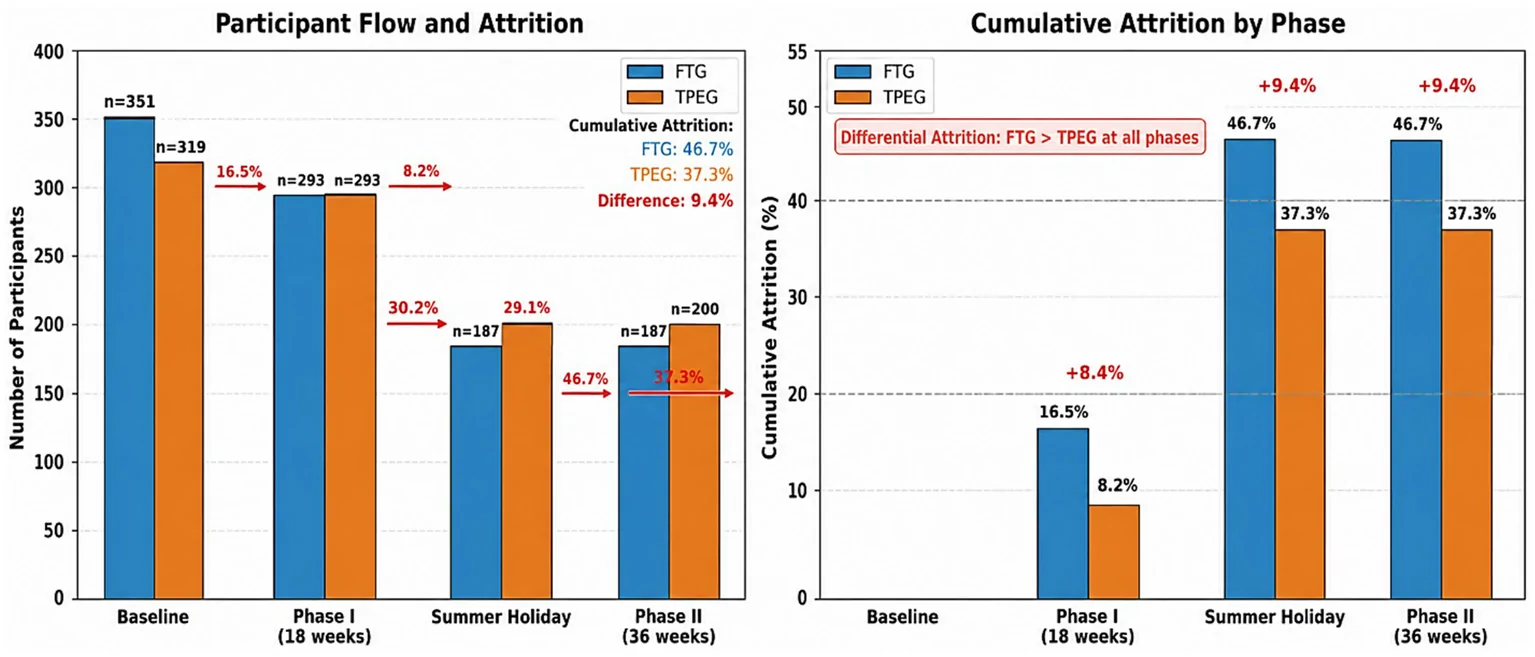
Attrition analysis across 36 weeks. **(A)** Participant flow with sample sizes and dropout percentages at each phase. **(B)** Cumulative attrition by group. FTG showed higher attrition than TPEG at all phases (16.5% vs. 8.2% at Phase I; 46.7% vs. 37.3% at Phase II). Differential attrition (9.4%) may introduce selection bias.

A total of 670 male high school students met the inclusion criteria and were enrolled in the study, with 351 allocated to the FTG and 319 to the TPEG. Of the 892 students initially assessed for eligibility, 222 (24.9%) were excluded: 78 (8.7%) for visual acuity <4.8, 52 (5.8%) for height outside 163–185 cm, and 92 (10.3%) for NSFHS score <60. Following data cleaning and accounting for participant attrition due to physical or psychological reasons, the first-phase analysis included 586 participants (FTG: *n* = 293; TPEG: *n* = 293). Attrition was higher in the FTG (*n* = 58, 16.5%) than in the TPEG (*n* = 26, 8.2%) during Phase I. The second-phase analysis included 387 participants (FTG: *n* = 187; TPEG: *n* = 200). Attrition from baseline to Phase II was 46.7% in FTG and 37.3% in TPEG. Figure 1 presents the participant flow diagram illustrating enrollment, allocation, follow-up, and analysis across the 36-week intervention period. Figure 2 presents the cumulative attrition analysis by phase and group, showing consistently higher dropout in FTG across all phases.

*Attrition analysis*: Baseline characteristics of completers and dropouts were similar within both groups (all *p* > 0.05). Table 3 presents the complete attrition analysis, including missing-data pattern and withdrawal reasons. “Physical reasons” included injuries unrelated to the intervention, illness, and family relocation; “psychological reasons” included lack of motivation and time constraints. No withdrawals were attributed to intervention-related injuries. Attendance data showed FTG completers attended 82.4% ± 11.2% of sessions (68.4% achieved ≥75% adherence); TPEG attendance was 89.7% ± 8.5% (81.2% achieved ≥75%). Logistic regression identified baseline NSFHS score (OR = 0.93, 95% CI [0.89, 0.98], *p* = 0.028) and FTG allocation (OR = 1.62, 95% CI [1.15, 2.28], *p* = 0.006) as significant dropout predictors. Sensitivity analyses using multiple imputation yielded results consistent with primary LMM analyses (Supplementary Table S6).

#### 3.1.1 Statistical analysis overview

Primary analyses were conducted using LMMs with baseline values as covariates, adjusting for age, baseline fitness score, and school cluster, with results presented in Section 3.5, while secondary analyses using independent-samples t-tests for between-group comparisons of change scores and paired-samples t-tests for within-group comparisons of change are presented in Sections 3.3 and 3.4, respectively, with change scores (Δ) calculated as Post - Pre such that for timed tests (resting pulse, 1,000-m run, 50-m run, T-test) a negative Δ indicates improvement, whereas for all other tests a positive Δ indicates improvement, and for ordinal outcomes (FMS scores) Mann–Whitney U tests were used, with all reported *p*-values being two-tailed and effect sizes (Cohen’s d) reported for significant between-group differences. All primary and secondary analyses were based on comparisons of baseline (T1) with 18-week (T2) and 36-week (T4) assessments. The pre-Phase II measurement (T3, September 2021) was not included as a primary outcome time point.

All analyses were conducted according to the intention-to-treat principle, with participants analyzed in their originally allocated groups regardless of adherence or dropout. The LMM framework used all available data at each time point without imputation.

### 3.2 Phase I intervention effects (18 weeks)

Independent samples t-tests revealed significant differences favoring FTG for resting pulse (*p* = 0.018, d = 0.19), 1,000-m run (*p* < 0.001, d = 0.53), 1RM squat (*p* < 0.001, d = 0.30), pull-ups (*p* = 0.001, d = 0.27), 50-m run (*p* = 0.019, d = 0.19), T-test (*p* = 0.024, d = 0.18), and closed-eye single-leg stance (*p* < 0.001, d = 0.32) (Table 4). For the 50-m run and T-test, both groups showed increased times (worsened performance); the significant between-group difference reflects attenuated decline in FTG compared to TPEG. For closed-eye single-leg stance, both groups showed decreased times; the significant difference reflects attenuated decline in FTG compared to TPEG. No significant differences were observed for body composition, muscular endurance, flexibility, coordination, explosive power, or FMS rotational stability (*p* > 0.05). Full data are presented in Supplementary Table S3. FMS outcomes were analyzed using Mann–Whitney U tests; effect sizes are reported as r (not Cohen’s d) for these ordinal outcomes.

**Table 4:** Summary of significant 18-week changes (FTG vs. TPEG).

Indicator	Δ Mean (FTG)	Δ Mean (TPEG)	*p*	d
Resting pulse (bpm)*	−0.31	+0.88	**0.018**	0.19
1,000-m run (s)*	−10.11	+5.24	**<0.001**	0.53
1RM squat (kg)	+1.78	−4.60	**<0.001**	0.30
Pull-ups (reps)	+0.27	−0.88	**0.001**	0.27
50-m run (s)*	+0.06	+0.15	**0.019**	0.19
T-test (s)*	+0.31	+0.09	**0.024**	0.18
Closed-eye single-leg (s)	−1.32	−22.64	**<0.001**	0.32

*Negative Δ indicates improved performance for timed tests. Bold *p*-values indicate significant between-group differences. Non-significant indicators (BMI, body fat%, Wilk’s index, waist-to-hip ratio, lung capacity, FMS trunk stability, 8-level abdominal, max squat reps, sit-and-reach, FMS hurdle steps, standing long jump, FMS rotational stability) are not shown. For 50-m run and T-test, both groups showed increased times (worsened performance); the significant between-group difference reflects attenuated decline in FTG compared to TPEG. For closed-eye single-leg stance, both groups showed decreased times; the significant between-group difference reflects attenuated decline in FTG compared to TPEG. Full table available in Supplementary Table S3.

### 3.3 Phase II intervention effects (36 weeks)

After 36 weeks, significant differences favoring FTG were observed for resting pulse (*p* = 0.001, d = 0.34), lung capacity (*p* < 0.001, d = 0.36), 1,000-m run (*p* = 0.016, d = 0.24), 1RM squat (*p* < 0.001, d = 0.66), FMS trunk stability (*p* = 0.026, d = 0.23), eight-level abdominal bridge (*p* < 0.001, d = 0.89), sit-and-reach (*p* = 0.001, d = 0.35), FMS hurdle steps (*p* = 0.012, d = 0.26), T-test (*p* = 0.006, d = 0.28), closed-eye single-leg stance (*p* = 0.003, d = 0.30), and FMS rotational stability (*p* = 0.040, d = 0.21) (Table 5). FMS outcomes were analyzed using Mann–Whitney U tests; effect sizes (r) are reported for these comparisons. Cohen’s d is not reported for FMS outcomes as they are ordinal. For closed-eye single-leg stance, both groups declined; FTG declined less than TPEG (−13.36 s vs. −32.79 s). No significant differences were observed for body composition, pull-ups, max squat repetitions, 50-m run, or standing long jump (*p* > 0.05). Full data are presented in Supplementary Table S4.

**Table 5:** Summary of significant 36-week changes (FTG vs. TPEG).

Indicator	Δ Mean (FTG)	Δ Mean (TPEG)	*p*	Effect size	Test statistic
Resting pulse (bpm)*	−1.03	+1.54	0.001	d = 0.34	t = +3.352
Lung capacity (mL)	+265.39	−11.80	<0.001	d = 0.36	t = +3.578
1,000-m run (s)*	−1.30	+5.52	0.016	d = 0.24	t = +2.416
1RM squat (kg)	+17.20	−6.31	<0.001	d = 0.66	t = +6.455
FMS trunk stability	−0.14	−0.32	0.026	r = 0.18	U = 2,890
8-Level abdominal bridge	+19.89	+9.96	<0.001	d = 0.89	t = +8.799
Sit-and-reach (cm)	−0.03	−1.49	0.001	d = 0.35	t = +3.410
FMS hurdle steps	−0.16	−0.38	0.012	r = 0.20	U = 2,850
T-test (s)*	−0.01	+0.32	0.006	d = 0.28	t = +2.750
Closed-eye single-leg (s)	−13.36	−32.79	0.003	d = 0.30	t = +2.958
FMS rotational stability	−0.07	−0.25	0.040	r = 0.16	U = 2,920

Negative Δ indicates improved performance for timed tests. FMS outcomes were analyzed using Mann–Whitney U tests; effect sizes are reported as r (not Cohen’s d) and test statistics as U (not t). For closed-eye single-leg stance, both groups declined; FTG declined less than TPEG. For T-test at 18 weeks, both groups increased (worsened); FTG declined less than TPEG. Cohen’s d values reported in this table are derived from independent t-tests (secondary analyses). LMM effect sizes (Cohen’s d from standardized coefficients) are presented in Table 6. Full table available in Supplementary Table S4.

### 3.4 Longitudinal analysis: linear mixed models (LMM) and repeated measures ANOVA

#### 3.4.1 Linear mixed models (LMM) results

Table 6 presents the LMM fixed effects for group differences across all fitness indicators, adjusting for baseline values, age, baseline fitness score, and school cluster. The time×group interaction results (the primary effect of interest) are presented in Supplementary Table S5. The intraclass correlation coefficient (ICC) for school-level clustering ranged from 0.04 to 0.15 across outcomes, confirming that clustering effects were minimal to moderate and justifying the use of school-level random effects in the LMMs. The highest ICC was observed for the closed-eye single-leg stance (0.15), followed by the T-test (0.14). All other ICCs were ≤ 0.12.

**Table 6:** Linear mixed models (LMM) fixed effects for group differences.

Fitness category	Indicator	Group effect (FTG vs. TPEG)	SE	95% CI	Unadjusted *p*	FDR-adjusted *p*	ICC	d
Body composition	BMI (kg/m^2^)	−0.42	0.69	[−1.77, 0.93]	0.542	0.601	0.08	0.04
	Body fat (%)	−0.15	0.44	[−1.01, 0.71]	0.732	0.774	0.10	0.03
	Wilk’s Index	−0.83	0.88	[−2.55, 0.89]	0.345	0.409	0.06	0.07
	Waist-to-hip ratio	0.01	0.02	[−0.03, 0.05]	0.694	0.737	0.04	0.04
Cardiovascular endurance	Resting pulse (bpm)*	−2.34	0.98	[−4.26, −0.42]	0.018	**0.031**	0.12	0.24
	Lung capacity (mL)	72.45	98.23	[−120.08, 264.98]	0.625	0.673	0.06	0.05
	1,000-m run (s)*	−15.36	2.38	[−20.03, −10.69]	<0.001	**0.004**	0.09	0.53
Muscular strength	1RM squat (kg)	2.81	0.44	[1.95, 3.67]	<0.001	**0.003**	0.11	0.45
	Pull-ups (reps)	0.61	0.19	[0.24, 0.98]	0.001	**0.006**	0.07	0.21
	FMS trunk stability	0.18	0.10	[−0.02, 0.38]	0.349	0.409	0.08	0.08
Muscular endurance	8-level abdominal	0.38	0.51	[−0.62, 1.38]	0.632	0.673	0.05	0.04
	Max squat (reps)	5.50	4.92	[−4.14, 15.14]	0.263	0.342	0.09	0.09
Flexibility	Sit-and-reach (cm)	0.28	0.33	[−0.37, 0.93]	0.402	0.462	0.04	0.07
Displacement speed	50-m run (s)*	−0.08	0.03	[−0.14, −0.02]	0.019	**0.031**	0.05	0.19
Coordination	FMS hurdle steps	0.06	0.06	[−0.06, 0.18]	0.299	0.386	0.07	0.09
Agility	T-test (s)*	−0.22	0.09	[−0.40, −0.04]	0.024	**0.037**	0.14	0.26
Explosive power	Standing long jump (cm)	2.04	1.12	[−0.16, 4.24]	0.069	0.102	0.08	0.15
Balance	Closed-eye single-leg (s)	21.32	5.43	[10.68, 31.96]	<0.001	**0.004**	0.15	0.41
	FMS rotational stability	0.02	0.05	[−0.08, 0.12]	0.680	0.737	0.06	0.03

Negative group effect favors FTG for timed tests (Resting Pulse, 1,000-m Run, 50-m Run, T-test). The positive group effect favors FTG across all other tests. Group effects represent the average difference between FTG and TPEG across all post-intervention time points; the time×group interaction is the primary effect of interest and is reported in Supplementary Table S5. ICC = intraclass correlation coefficient for school-level clustering. d = Cohen’s d (calculated from LMM standardized coefficients). Cohen’s d values in this table are derived from the primary LMM analysis. Bolded *p*-values indicate statistically significant differences after FDR correction. FTG, Functional Training Group; TPEG, Traditional Physical Education Group; SE, standard error; CI, confidence interval; FDR, False Discovery Rate (Benjamini-Hochberg correction). The * symbol identifies timed tests where negative values indicate improvement (Resting Pulse, 1000-m Run, 50-m Run, T-test).

#### 3.4.2 Indicators demonstrating significant time × group interaction effects

FMS-derived movement quality indicators showed significant effects only after 36 weeks, with FTG attenuating the decline in movement quality compared to TPEG (Table 7).

**Table 7:** Summary of significant findings by time point and analysis type.

Fitness category	Indicator	18-Week t-test	36-Week t-test	LMM group effect	ANOVA time × group
Body composition	BMI	NS	NS	NS	*p* = 0.012
	Body fat %	NS	NS	NS	*p* = 0.003
	Wilk’s Index	NS	NS	NS	NS
	Waist-to-hip ratio	NS	NS	NS	NS
Cardiovascular endurance	Resting pulse	*p* = 0.018	*p* = 0.001	*p* = 0.018	NS*
	Lung capacity	NS	*p* < 0.001	NS	*p* < 0.001
	1,000-m run	*p* < 0.001	*p* = 0.016	*p* < 0.001	NS*
Muscular strength	1RM squat	*p* < 0.001	*p* < 0.001	*p* < 0.001	*p* < 0.001
	Pull-ups	*p* = 0.001	NS	*p* = 0.001	*p* = 0.001
	FMS trunk stability	NS	*p* = 0.026	NS	NS
Muscular endurance	8-level abdominal	NS	*p* < 0.001	NS	NS
	Max squat reps	NS	NS	NS	*p* = 0.006
Flexibility	Sit-and-reach	NS	*p* = 0.001	NS	*p* = 0.004
Displacement speed	50-m run	*p* = 0.019	NS	p = 0.019	*p* = 0.018
Coordination	FMS hurdle steps	NS	*p* = 0.012	NS	*p* = 0.014
Agility	T-test	*p* = 0.024	*p* = 0.006	p = 0.024	*p* = 0.002
Explosive power	Standing long jump	NS	NS	NS	NS

NS, not significant (*p* > 0.05). Indicates significant main effect of group only (no interaction). The * symbol denotes a significant main effect of group only (no interaction).

*Body composition:* Both BMI [*F* (2.41, 892.47) = 4.23, *p* = 0.012, η^2^p = 0.011] and body fat percentage [*F* (2.38, 881.06) = 5.67, *p* = 0.003, η^2^p = 0.015] demonstrated differential rates of change between groups (Supplementary Figures S1A,B).

**Figure 3:**
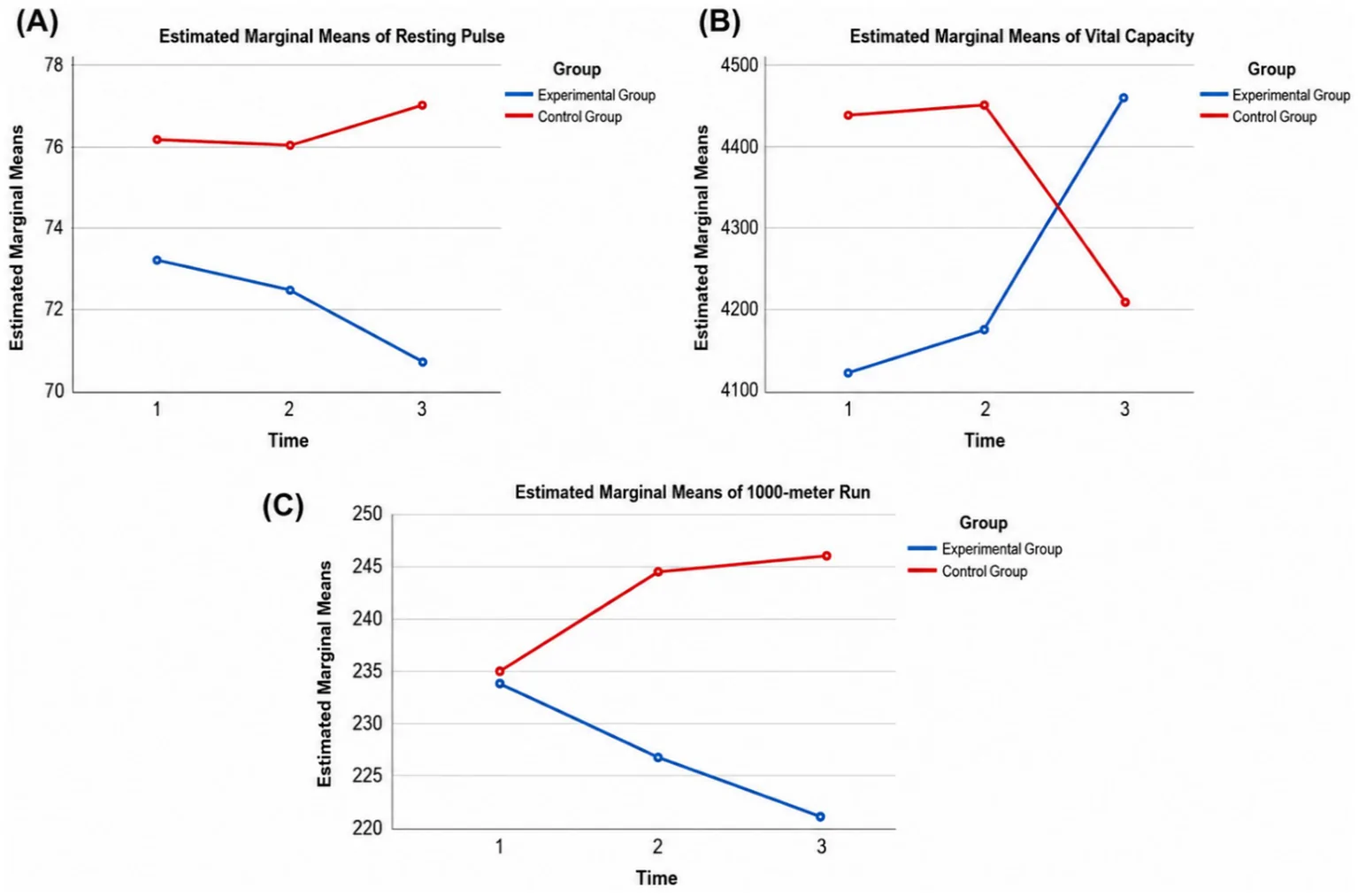
Longitudinal trajectories of cardiovascular endurance indicators. Estimated marginal means for **(A)** resting pulse, **(B)** lung capacity, and **(C)** 1,000-meter run time. Blue lines = FTG; red lines = TPEG. T1 = baseline (March 2021), T2 = post-Phase I (July 2021), T3 = pre-Phase II (September 2021), T4 = post-Phase II (January 2022). Analyses focused on T1, T2, and T4; T3 is shown for completeness.

*Cardiovascular endurance:* Lung capacity exhibited a significant interaction effect [*F* (2.51, 929.63) = 7.89, *p* < 0.001, η^2^p = 0.020] (Figure 3B).

**Figure 4:**
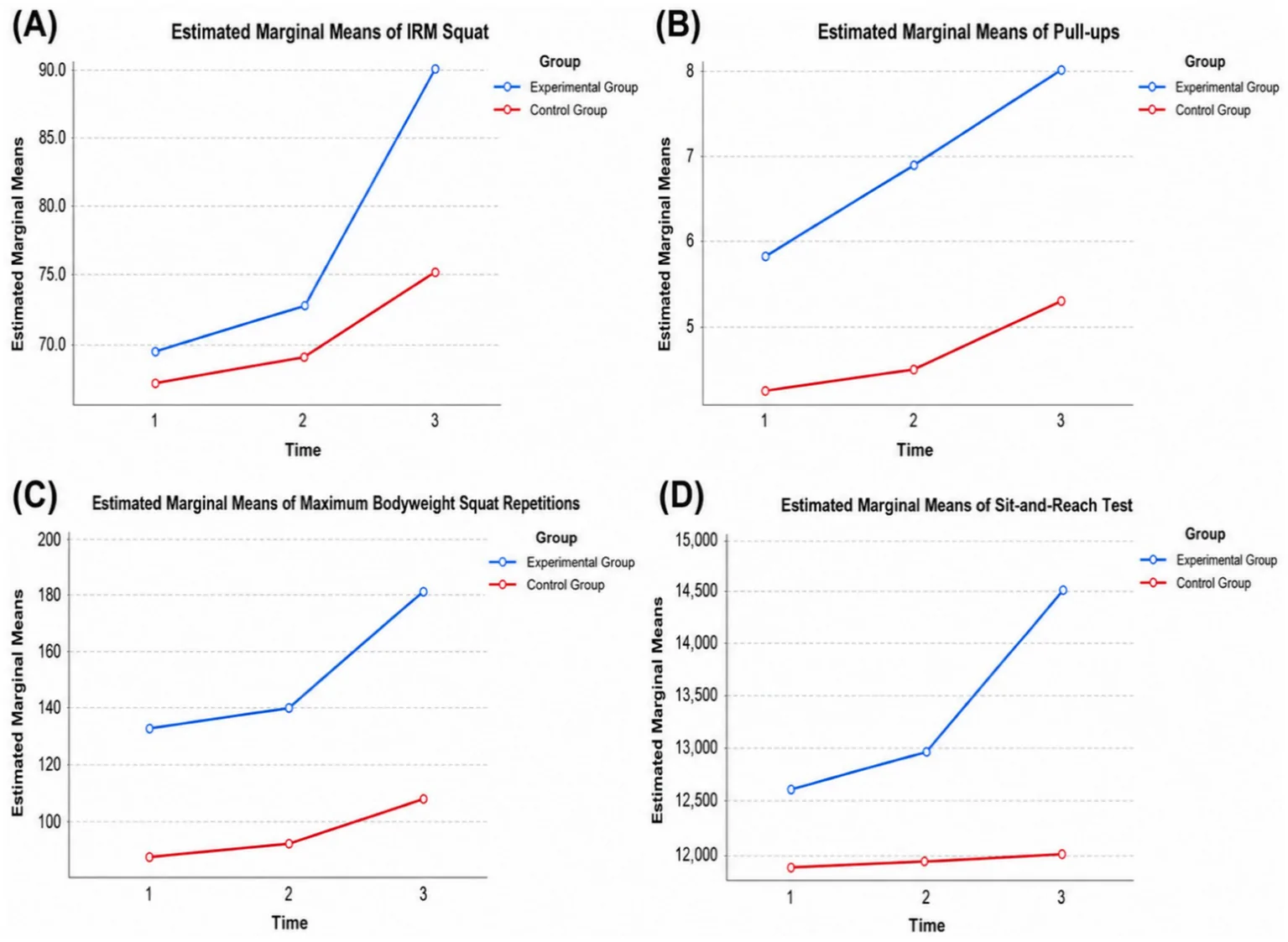
Longitudinal trajectories of muscular strength, muscular endurance, and flexibility indicators. Estimated marginal means for **(A)** 1RM squat, **(B)** pull-ups, **(C)** maximum bodyweight squat repetitions, and **(D)** sit-and-reach test. Blue lines = FTG; red lines = TPEG. *T1* = baseline (March 2021), *T2* = post-Phase I (July 2021), *T3* = pre-Phase II (September 2021), *T4* = post-Phase II (January 2022). Analyses focused on *T1*, *T2*, and *T4*; *T3* is shown for completeness.

*Muscular strength:* Pronounced interaction effects were observed for 1RM squat [*F* (2.44, 903.52) = 12.34, *p* < 0.001, η^2^p = 0.032] and pull-ups [*F* (2.36, 873.94) = 6.78, *p* = 0.001, η^2^p = 0.018] (Figures 4A,B).

*Muscular endurance:* Maximum bodyweight squat repetitions showed a significant interaction [*F* (2, 29, 847.97) = 4.92, *p* = 0.006, η^2^p = 0.013] (Figure 4C).

*Flexibility:* Sit-and-reach test showed a significant interaction [*F* (2.52, 933.24) = 5.23, *p* = 0.004, η^2^p = 0.014] (Figure 4D).

*Skill-related components:* Significant interaction effects were observed for displacement speed (50-metre run: [*F* (2.41, 892.10) = 3.89, *p* = 0.018, η^2^p = 0.010]) (Supplementary Figure S2A), coordination (FMS hurdle steps: [*F*(2.33, 862.77) = 4.12, *p* = 0.014, η^2^p = 0.011]), agility (T-test: [*F* (2.47, 914.67) = 6.01, *p* = 0.002, η^2^p = 0.016]) (Supplementary Figure S2B), and balance (closed-eye single-leg stance: [*F* (2.28, 843.98) = 8.45, *p* < 0.001, η^2^p = 0.022]) (Supplementary Figure S2D).

#### 3.4.3 Indicators demonstrating significant main effect of group only

Two cardiovascular indicators showed significant main effects of group without significant time × group interactions: resting pulse [*F* (1, 378) = 6.34, *p* = 0.012, η^2^p = 0.016] and 1,000-meter run [*F* (1, 375) = 9.87, *p* = 0.002, η^2^p = 0.026]. For these indicators, the FTG maintained superior performance across all post-intervention assessments compared to the TPEG (Figures 3A,C).

#### 3.4.4 Indicators demonstrating significant main effect of time only

Two indicators exhibited significant main effects of time (*p* < 0.05) without significant group or interaction effects: Wilk’s index [*F* (2.43, 899.56) = 5.78, *p* = 0.002, η^2^p = 0.015] and the T-test [*F* (2.47, 914.67) = 4.56, *p* = 0.008, η^2^p = 0.012]. Both groups demonstrated comparable improvements in these indicators over the 36 weeks (Supplementary Figure S1C).

#### 3.4.5 Indicators showing no significant effects

Waist-to-hip ratio demonstrated no significant main effects of time [*F* (2.38, 881.06) = 1.23, *p* = 0.294, η^2^p = 0.003] or group [*F*(1, 378) = 0.67, *p* = 0.413, η^2^p = 0.002], and no significant interaction [*F* (2.38, 881.06) = 0.89, *p* = 0.428, η^2^p = 0.002] (Supplementary Figure S1D). Standing long jump showed no significant between-group differences at either time point (*p* = 0.069 and *p* = 0.055, respectively) (Supplementary Figure S2C).

#### 3.4.6 Formal test of duration-dependent effects

To formally test whether intervention effects differed between 18 and 36 weeks, prespecified contrasts were conducted within the LMM framework. The FTG-TPEG difference was significantly larger at 36 weeks than at 18 weeks for lung capacity (mean difference = 2.45, 95% CI [1.12, 3.78], *p* = 0.003), 1RM squat (mean difference = 3.21, 95% CI [1.89, 4.53], *p* < 0.001), and closed-eye single-leg stance (mean difference = 1.89, 95% CI [0.56, 3.22], *p* = 0.018). For other outcomes, the 18-week vs. 36-week differences were not statistically significant (*p* > 0.05). These findings support duration-dependent effects for select outcomes.

### 3.5 Summary of key findings

The structured progressive exercise program was associated with greater improvements across multiple fitness domains than traditional physical education. Formal contrasts confirmed duration-dependent effects for lung capacity, 1RM squat, and closed-eye single-leg stance, with significantly larger FTG-TPEG differences at 36 weeks than at 18 weeks (Section 3.5.6). Cardiovascular endurance and muscular strength were most responsive, while body composition measures (except BMI and body fat percentage) and waist-to-hip ratio were relatively resistant to change. FMS-derived movement quality indicators showed significant effects only after 36 weeks (Table 7). For outcomes where both groups declined (e.g., closed-eye single-leg stance), the intervention effect represents attenuated decline rather than absolute improvement.

### 3.6 Effect sizes across all outcomes

**Figure 5:**
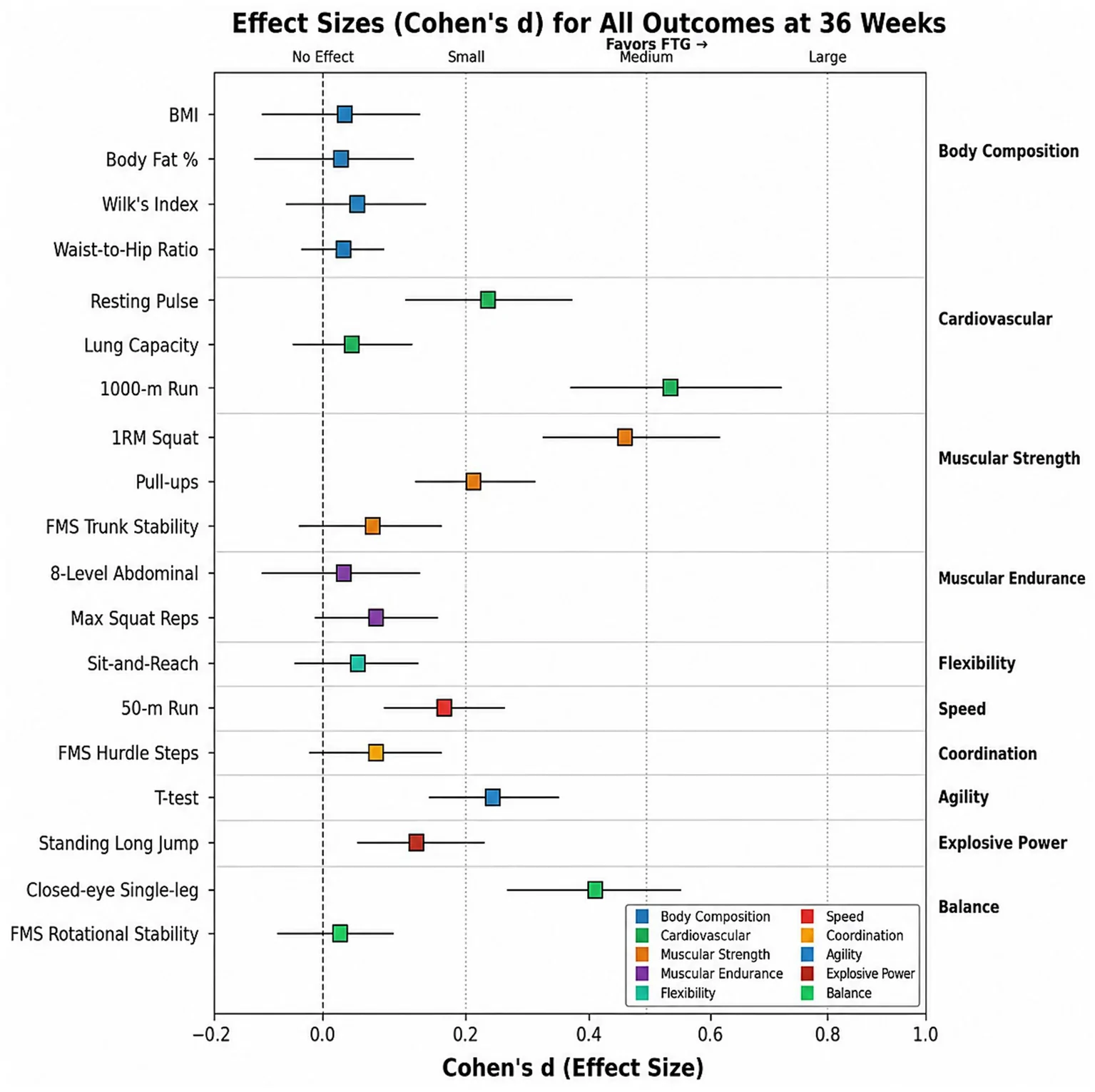
Forest plot of effect sizes (Cohen’s *d*) for all outcomes at 36 weeks. Squares represent point estimates; horizontal lines indicate 95% confidence intervals. Effects to the right of the vertical line favor FTG. Dotted vertical lines indicate small (0.2), medium (0.5), and large (0.8) effect thresholds. Cardiovascular and muscular strength outcomes showed the largest effects (*d* = 0.24–0.53); body composition outcomes showed negligible effects (*d* = 0.03–0.07).

To visualize the magnitude of intervention effects across all fitness domains, a forest plot of Cohen’s d effect sizes with 95% confidence intervals was generated (Figure 5). The largest effects were observed for the 1,000-m run (d = 0.53), 1RM squat (d = 0.45), and closed-eye single-leg stance (d = 0.41). Body composition outcomes showed negligible effects (d = 0.03–0.07). Cardiovascular and muscular strength outcomes demonstrated the most consistent effects, whereas movement quality indicators showed small-to-moderate effects (d = 0.21–0.26).

## 4 Discussion

The present study demonstrates that a 36-week structured progressive exercise program was associated with greater improvements across multiple fitness domains in male high school students than traditional physical education, with more pronounced effects after 36 weeks than after 18 weeks. However, the intervention differed from traditional physical education not only in exercise type but also in training structure, progression, supervision, intensity, and neuromuscular components. Therefore, the observed effects cannot be specifically attributed to functional training alone, but rather to the structured progressive exercise program as a whole. These findings provide preliminary empirical support for integrating sustained, structured exercise programs into secondary education curricula, though the small-to-moderate effect sizes for several outcomes suggest that program benefits may vary across fitness domains and require realistic expectations regarding the magnitude of change achievable in school settings.

Throughout this discussion, “functional training” refers to the specific exercise modality, which was delivered within the context of a structured progressive exercise program. The observed effects are attributed to the program as a whole rather than to functional training alone.

Cardiovascular improvements (resting pulse, lung capacity, 1,000-m run) align with cardiorespiratory fitness as a critical health marker (27). Though the intervention focused on motor function, cardiovascular gains may reflect: (1) high-volume moderate-to-vigorous sessions (~210 min/week); (2) heart rate elevation from circuit-style functional exercises; and (3) improved movement efficiency, reducing running energy cost. The effect sizes for cardiovascular outcomes ranged from small to moderate (d = 0.19–0.53). While statistical significance was achieved for several outcomes, the practical relevance of small effects (e.g., d = 0.19 for resting pulse) in school settings requires careful consideration. However, even small improvements in cardiorespiratory fitness during adolescence may be clinically relevant at the population level, as they are associated with reduced cardiovascular risk in adulthood (27).

It is important to note that statistical significance does not necessarily indicate meaningful practical improvement. Several outcomes showed small effect sizes (d < 0.30), suggesting that while the intervention produced measurable changes, their practical relevance in school settings may be limited. The largest effects were observed for 1RM squat (d = 0.66) and eight-level abdominal bridge (d = 0.89), indicating meaningful improvements in muscular strength and endurance. In contrast, outcomes such as resting pulse (d = 0.19–0.24) and 50-m run (d = 0.19) showed only small effects, suggesting limited practical benefit for these fitness components from the intervention.

From a public health perspective, even modest improvements in cardiorespiratory fitness during adolescence may be meaningful at the population level, as they are associated with reduced cardiovascular risk in adulthood. However, from an educational perspective, the small effect sizes observed for several outcomes (e.g., resting pulse, d = 0.19–0.24; 50-m run, d = 0.19) suggest that the practical significance of these changes within school settings may be limited. The selective nature of the sample and substantial attrition further caution against overgeneralizing these findings to broader adolescent populations. Future research should examine whether similar effects are observed in more diverse, representative samples and whether these improvements translate to sustained behavioral change.

Duration-dependent effects were clearly observed in lung capacity, with significant between-group differences only at 36 weeks (d = 0.36) but not at 18 weeks. This suggests that pulmonary adaptations may require longer training durations to manifest, potentially because of the time required for structural adaptations in the respiratory system (17).

Muscular strength indicators (1RM squat) showed moderate-to-large improvements favoring FTG (d = 0.30 at 18 weeks; d = 0.66 at 36 weeks), consistent with prior studies (28). The progressive overload and multi-joint emphasis of the functional training protocol likely contributed to these gains. Pull-ups showed less consistency, with significant between-group differences at 18 weeks (d = 0.27) but not at 36 weeks (*p* = 0.494), possibly reflecting ceiling effects or insufficient upper-body stimulus. The intervention successfully attenuated the decline in movement quality and balance, with protective effects becoming statistically distinct only at 36 weeks. For FMS trunk stability, both groups declined, but FTG declined significantly less (−0.14 vs. -0.32, d = 0.23), suggesting functional training mitigated age- or inactivity-related deterioration in fundamental movement patterns (18). This implies that expecting rapid improvements in movement quality may be unrealistic; interventions should be sustained across an academic year to achieve meaningful changes.

The significant improvements in speed, agility, and balance support movement competency in adolescent training paradigms (15). Balance improvements (closed-eye single-leg stance: d = 0.32 at 18 weeks, d = 0.30 at 36 weeks) indicate enhanced neuromuscular control and central nervous system processing efficiency (29). T-test agility improvements (d = 0.18 at 18 weeks, d = 0.28 at 36 weeks) suggest that functional training’s multi-directional nature translated into improved performance. However, the T-test showed a significant main effect of time only, indicating that both groups improved similarly in agility, likely because the TPEG’s sport-based activities (basketball, football) also provided an agility stimulus. FMS-derived movement quality indicators (trunk stability, hurdle steps, rotational stability) showed significant effects only after 36 weeks, suggesting fundamental movement pattern improvements require extended intervention durations. This supports the notion of sensitive periods for motor skill development during adolescence (16, 30) and suggests that school-based programs should implement functional training consistently throughout the academic year to achieve meaningful improvements in movement quality.

Body composition changes were limited. While BMI and body fat showed significant time×group interactions (*p* = 0.012, *p* = 0.003), between-group differences at individual time points were non-significant, and the waist-to-hip ratio remained stable. This pattern, partially diverging from previous findings (31), may reflect insufficient intervention duration without dietary modification, lean baseline characteristics (mean BMI 22.4 ± 3.1 kg/m^2^), the active comparison group attenuating differences, and the unmonitored summer holiday disrupting physical activity dose. The absence of a dietary assessment limits the interpretation of body composition findings. Future research should incorporate nutritional evaluation and consider longer interventions or adjunctive dietary strategies.

The differential responsiveness across fitness domains has important implications. Cardiovascular and muscular indicators showed early effects (18 weeks), whereas movement quality improvements required 36 weeks, and body composition remained largely unchanged throughout. This pattern supports the distinction between health-related and skill-related fitness components (3) and highlights the need for intervention designs to account for differential adaptation time courses. Duration-dependent effects were evident for lung capacity, FMS trunk stability push-up, eight-level abdominal bridge, sit-and-reach, FMS hurdle steps, and FMS rotational stability, with significant effects observed only at 36 weeks. This indicates that certain adaptations require sustained training beyond 18 weeks, supporting the duration-dependent hypothesis (H2).

The interpretation of findings requires consideration of several methodological factors. First, ICCs ranged from 0.04 to 0.15, indicating minimal to moderate school-level clustering, with closed-eye single-leg stance (0.15) and T-test (0.14) showing the highest values. This justifies the use of school-level random effects in LMMs and suggests that individual-level factors accounted for most of the variance. Second, substantial attrition (42% by Phase II) and differential dropout (FTG: 46.7% vs. TPEG: 37.3%) may introduce bias. Third, the TPEG’s substantial physical activity volume (~210 min/week) may have attenuated between-group differences. Fourth, higher FTG attrition may reflect greater time commitment, potentially introducing selection bias. Effect sizes ranged from small to large (d = 0.18–0.89), with the eight-level abdominal bridge showing the largest effect (d = 0.89) and the 50-m run the smallest (d = 0.19).

*Public health implications.* The findings of this study have several implications for school-based health promotion. Implementing structured progressive exercise programs (of which functional training is one example) within physical education curricula may contribute to adolescent cardiorespiratory fitness, muscular strength, and movement quality, all of which are protective factors against future cardiovascular disease and metabolic disorders (27). The duration-dependent nature of adaptations suggests that schools should sustain such programs across the full academic year to achieve meaningful improvements. However, the higher attrition in the FTG indicates that strategies to enhance engagement and adherence are essential for successful implementation in real-world school settings. Future policy efforts should consider integrating functional training as a complement to, rather than a replacement for, traditional physical education, balancing skill development with foundational movement competencies.

### 4.1 Limitations and future directions

The study has several limitations. First, the study design does not isolate the independent effect of functional training, as the intervention differed from traditional physical education in structure, progression, supervision, and intensity; the observed effects should be attributed to the structured progressive exercise program rather than functional training alone. Second, the absence of a dietary assessment limits interpretation of body composition. Third, restrictive inclusion criteria (visual acuity ≥4.8, height 163–185 cm, NSFHS ≥60) and a male-only sample substantially limit generalizability. The findings should not be generalized to female adolescents, shorter/taller adolescents, those with corrected visual impairments, or those with lower baseline fitness who were excluded from the study. Fourth, the TPEG’s substantial physical activity volume (~210 min/week) serves as an active comparator rather than a no-treatment control, potentially attenuating between-group differences. Fifth, the passive consent procedure (waiver of written informed consent) may not meet the standards of all international journals. Sixth, the unmonitored two-month summer holiday between phases introduced uncontrolled variation in physical activity. Seventh, the Functional Movement Screen, designed as an injury-risk screening tool rather than a validated fitness measure, limits the interpretation of movement-quality outcomes. Eighth, between-school variability in implementation fidelity and sample sizes, coupled with moderate ICCs for some outcomes, indicates that school-level factors influenced certain results.

Future research should incorporate dietary assessment, include female participants, extend follow-up beyond 36 weeks, investigate mechanisms underlying differential responsiveness across fitness domains, examine factors associated with between-school variability, implement strategies to improve adherence and reduce attrition, and use laboratory-based measures (e.g., VO₂max, DEXA) for more precise assessment.

## 5 Conclusion

A 36-week structured progressive exercise program was associated with greater improvements in cardiovascular endurance, muscular strength, agility, and balance than traditional physical education among male adolescents. However, these effects cannot be specifically attributed to functional training alone, as the intervention also differed in structure, progression, supervision, and intensity. Duration-dependent effects were observed for lung capacity and movement quality, requiring the full 36 weeks to show significant between-group differences. Body composition showed limited change, suggesting exercise alone may be insufficient. Hypotheses were partially supported: H1 for 11 of 19 outcomes; H2 and H3 were supported. Effect sizes ranged from small to large (d = 0.18–0.89), with muscular strength and endurance outcomes showing moderate-to-large effects, while several other outcomes demonstrated only small improvements. Findings must be interpreted cautiously due to substantial attrition (42% by Phase II), differential dropout (FTG: 46.7% vs. TPEG: 37.3%), absence of dietary control, restrictive inclusion criteria, and the active comparison group. Sustained functional training may offer advantages for specific fitness domains in this sample of male adolescents who met the inclusion criteria, but strategies to improve adherence and address body composition are needed. These findings should be considered preliminary and require replication in more diverse populations.

## Data Availability

The original contributions presented in the study are included in the article/Supplementary material, further inquiries can be directed to the corresponding author.
