# Supplementary material for: Comparison of a 36-week functional training program versus traditional physical education on physical fitness in male adolescents: a longitudinal school-based study
Source: Front Public Health. 2026 Sep 8;14:1936635. doi: 10.3389/fpubh.2026.1936635 (PMC13593854; doi:10.3389/fpubh.2026.1936635)
Supplement: Supplementary file 1 [file Data_Sheet_1.DOCX]

**Figure S1. Longitudinal trajectories of body composition indicators. Estimated marginal means for** (A) BMI, (B) body fat percentage, (C) Wilk's index, and (D) waist-to-hip ratio. Blue lines = FTG; red lines = TPEG. T1 = baseline (March 2021), T2 = post-Phase I (July 2021), T3 = pre-Phase II (September 2021), T4 = post-Phase II (January 2022). Analyses focused on T1, T2, and T4; T3 is shown for completeness.

**Figure S2. Longitudinal trajectories of skill-related fitness indicators. Estimated marginal means for** (A) 50-metre run, (B) T-test, (C) standing long jump, and (D) closed-eye single-leg stance. Blue lines = FTG; red lines = TPEG. T1 = baseline (March 2021), T2 = post-Phase I (July 2021), T3 = pre-Phase II (September 2021), T4 = post-Phase II (January 2022). Analyses focused on T1, T2, and T4; T3 is shown for completeness.

**Table S1.** Distribution of Participants Across Participating Schools

| **School No.** | **School Name** | **FTG (n)** | **TPEG (n)** | **Total (n)** |
| --- | --- | --- | --- | --- |
| 1 | Chishui No. 1 High School | 24 | 22 | 46 |
| 2 | Daozhen Middle School | 30 | 22 | 52 |
| 3 | Luoyang No. 1 High School | 26 | 27 | 53 |
| 4 | Meitan Qiushi High School | 20 | 20 | 40 |
| 5 | Renhuai No. 1 High School | 40 | 40 | 80 |
| 6 | Mouping No. 1 High School | 7 | 8 | 15 |
| 7 | Huayang Middle School | 23 | 27 | 50 |
| 8 | Suiyang No. 1 High School | 5 | 5 | 10 |
| 9 | Tanghu High School | 23 | 13 | 36 |
| 10 | Tongzi No. 1 High School | 5 | 5 | 10 |
| 11 | Xishui No. 1 High School | 27 | 23 | 50 |
| 12 | Yuqing Middle School | 24 | 25 | 49 |
| 13 | Zheng'an No. 1 High School | 23 | 15 | 38 |
| 14 | Fenggang No. 1 High School | 15 | 17 | 32 |
| 15 | Zunyi Normal University Experimental School | 12 | 12 | 24 |
| 16 | Zunyi No. 1 High School | 12 | 14 | 26 |
| 17 | Nanbai High School | 11 | 17 | 28 |
| 18 | Zunyi No. 4 High School | 24 | 7 | 31 |
| Total |  | 351 | 319 | 670 |

*Note:* FTG = Functional Training Group; TPEG = Traditional Physical Education Group. School sample sizes ranged from 5 to 80 participants. Schools 8 and 10 (Suiyang No. 1 and Tongzi No. 1) had smaller enrollments because only five students met all inclusion criteria and provided consent in these schools. Within each school, students were individually allocated to FTG or TPEG.

**Table S2.** Variance Components and Intraclass Correlation Coefficients from Linear Mixed Models.

| **Fitness Category** | **Indicator** | **Residual Variance (σ²)** | **School-Level Variance (τ²)** | **ICC** |
| --- | --- | --- | --- | --- |
| **Body Composition** |  |  |  |  |
|  | BMI (kg/m²) | 1.56 | 0.13 | 0.08 |
|  | Body Fat (%) | 12.89 | 1.43 | 0.10 |
|  | Wilk's Index | 15.23 | 0.97 | 0.06 |
|  | Waist-to-Hip Ratio | 0.005 | 0.0002 | 0.04 |
| **Cardiovascular Endurance** |  |  |  |  |
|  | Resting Pulse (bpm) | 34.56 | 4.72 | 0.12 |
|  | Lung Capacity (mL) | 52,345.67 | 3,342.10 | 0.06 |
|  | 1000-m Run (s) | 412.78 | 40.85 | 0.09 |
|  | Muscular Strength |  |  |  |
|  | 1RM Squat (kg) | 98.45 | 12.17 | 0.11 |
|  | Pull-ups (reps) | 4.56 | 0.34 | 0.07 |
|  | FMS Trunk Stability Push-up | 0.52 | 0.05 | 0.08 |
|  | Muscular Endurance |  |  |  |
|  | 8-Level Abdominal Bridge | 95.67 | 5.03 | 0.05 |
|  | Max Bodyweight Squat (reps) | 3,256.78 | 322.12 | 0.09 |
| **Flexibility** |  |  |  |  |
|  | Sit-and-Reach (cm) | 14.78 | 0.62 | 0.04 |
|  | Displacement Speed |  |  |  |
|  | 50-m Run (s) | 0.23 | 0.01 | 0.05 |
|  | Coordination |  |  |  |
|  | FMS Hurdle Steps | 0.56 | 0.04 | 0.07 |
|  | Agility |  |  |  |
|  | T-test (s) | 1.89 | 0.31 | 0.14 |
|  | Explosive Power |  |  |  |
|  | Standing Long Jump (cm) | 145.67 | 12.67 | 0.08 |
| **Balance** |  |  |  |  |
|  | Closed-eye Single-leg Stance (s) | 3,124.56 | 551.39 | 0.15 |
|  | FMS Rotational Stability | 0.62 | 0.04 | 0.06 |

*Note:* ICC = intraclass correlation coefficient = τ² / (σ² + τ²), representing the proportion of total variance attributable to school-level clustering. ICC values ≤ 0.05 indicate negligible clustering; 0.05–0.15 indicate moderate clustering; > 0.15 indicate substantial clustering. The highest ICC was observed for closed-eye single-leg stance (0.15), followed by the T-test (0.14). All other ICCs were ≤ 0.12, indicating minimal school-level effects for most outcomes.

**Table S3.** Comparison of Fitness Changes Between Functional Training and Traditional PE Groups After 18-Week Intervention (Phase I)

| **Category** | **Indicator** | **Group** | **n** | **Δ Mean ± SD** | **t** | **p** | **Cohen's d** | **95% CI** |
| --- | --- | --- | --- | --- | --- | --- | --- | --- |
| **Body Composition** |  |  |  |  |  |  |  |  |
|  | BMI (kg/m²) | FTG | 293 | -0.24 ± 1.68 | -0.610 | 0.542 | 0.05 | [-0.21, 0.11] |
|  |  | TPEG | 293 | -0.19 ± 1.60 |  |  |  |  |
|  | Wilk's Index | FTG | 293 | -0.19 ± 3.15 | -0.799 | 0.425 | 0.07 | [-0.23, 0.10] |
|  |  | TPEG | 293 | -0.02 ± 3.08 |  |  |  |  |
|  | Body Fat (%) | FTG | 293 | -0.03 ± 2.57 | -0.343 | 0.732 | 0.03 | [-0.13, 0.09] |
|  |  | TPEG | 293 | -0.08 ± 2.43 |  |  |  |  |
|  | Waist-to-Hip Ratio | FTG | 293 | 0.00 ± 0.03 | -0.899 | 0.369 | 0.07 | [-0.01, 0.01] |
|  |  | TPEG | 293 | 0.00 ± 0.03 |  |  |  |  |
| **Cardiovascular Endurance** |  |  |  |  |  |  |  |  |
|  | Resting Pulse (bpm)* | FTG | 293 | -0.31 ± 6.12 | 2.368 | 0.018 | 0.19 | [0.04, 0.49] |
|  |  | TPEG | 293 | 0.88 ± 6.09 |  |  |  |  |
|  | Lung Capacity (mL) | FTG | 293 | -50.48 ± 380.56 | -0.489 | 0.625 | 0.04 | [-0.23, 0.14] |
|  | TPEG | 293 | -66.53 ± 412.99 |  |  |  |  |  |
|  | 1000-m Run (s)* | FTG | 290 | -10.11 ± 28.08 | 6.453 | <0.001 | 0.53 | [0.37, 0.69] |
|  |  | TPEG | 293 | 5.24 ± 29.32 |  |  |  |  |
| **Muscular Strength** |  |  |  |  |  |  |  |  |
|  | 1RM Squat (kg) | FTG | 293 | 1.78 ± 7.28 | +3.630 | <0.001 | 0.30 | [0.14, 0.46] |
|  |  | TPEG | 293 | -4.60 ± 11.15 |  |  |  |  |
|  | Pull-ups (reps) | FTG | 293 | 0.27 ± 2.21 | +3.236 | 0.001 | 0.27 | [0.10, 0.43] |
|  |  | TPEG | 293 | -0.88 ± 2.36 |  |  |  |  |
|  | FMS Trunk Stability | FTG | 293 | -0.06 ± 0.64 | -0.936 | 0.349 | 0.08 | [-0.24, 0.09] |
|  |  | TPEG | 293 | -0.12 ± 0.76 |  |  |  |  |
| **Muscular Endurance** |  |  |  |  |  |  |  |  |
|  | 8-Level Abdominal | FTG | 292 | -0.66 ± 8.76 | -0.479 | 0.632 | 0.04 | [-0.12, 0.20] |
|  |  | TPEG | 292 | -1.04 ± 10.52 |  |  |  |  |
|  | Max Squat (reps) | FTG | 293 | -2.61 ± 43.83 | -1.121 | 0.263 | 0.09 | [-0.07, 0.25] |
|  |  | TPEG | 293 | -8.11 ± 71.67 |  |  |  |  |
| **Flexibility** |  |  |  |  |  |  |  |  |
|  | Sit-and-Reach (cm) | FTG | 293 | 0.12 ± 3.97 | -0.839 | 0.402 | 0.07 | [-0.09, 0.23] |
|  |  | TPEG | 293 | -0.16 ± 4.09 |  |  |  |  |
| **Displacement Speed** |  |  |  |  |  |  |  |  |
|  | 50-m Run (s)* | FTG | 293 | 0.06 ± 0.52 | 2.351 | 0.019 | 0.19 | [0.04, 0.35] |
|  |  | TPEG | 292 | 0.15 ± 0.41 |  |  |  |  |
| **Coordination** |  |  |  |  |  |  |  |  |
|  | FMS Hurdle Steps | FTG | 293 | -0.19 ± 0.67 | 1.039 | 0.299 | 0.09 | [-0.26, 0.08] |
|  |  | TPEG | 293 | -0.13 ± 0.75 |  |  |  |  |
| **Agility** |  |  |  |  |  |  |  |  |
|  | T-test (s)* | FTG | 293 | 0.31 ± 1.17 | -2.261 | 0.024 | 0.18 | [0.02, 0.35] |
|  |  | TPEG | 293 | 0.09 ± 1.22 |  |  |  |  |
| **Explosive Power** |  |  |  |  |  |  |  |  |
|  | Standing Long Jump (cm) | FTG | 293 | -2.63 ± 15.15 | -1.824 | 0.069 | 0.15 | [-0.01, 0.31] |
|  |  | TPEG | 292 | -4.67 ± 11.65 |  |  |  |  |
| **Balance** |  |  |  |  |  |  |  |  |
|  | Closed-eye Single-leg (s) | FTG | 293 | -1.32 ± 34.29 | -3.925 | <0.001 | 0.32 | [0.16, 0.49] |
|  |  | TPEG | 292 | -22.64 ± 86.43 |  |  |  |  |
|  | FMS Rotational Stability | FTG | 293 | -0.11 ± 0.67 | -0.413 | 0.680 | 0.03 | [-0.19, 0.13] |
|  |  | TPEG | 293 | -0.13 ± 0.73 |  |  |  |  |

*Note: For timed tests (resting pulse, 1000-m run, 50-m run, T-test), negative Δ indicates improved performance. For all other tests, positive Δ indicates improved performance. FTG = Functional Training Group; TPEG = Traditional Physical Education Group; d = Cohen's d; CI = confidence interval. Bolded p-values indicate statistically significant differences (p < 0.05). Effect sizes are reported only for significant comparisons.

**Table S4.** Comparison of Fitness Changes Between Functional Training and Traditional PE Groups After 36-Week Intervention (Phase II)

| **Category** | **Indicator** | **Group** | **n** | **Δ Mean ± SD** | **t** | **p** | **Cohen's d** | **95% CI** |
| --- | --- | --- | --- | --- | --- | --- | --- | --- |
| Body Composition |  |  |  |  |  |  |  |  |
|  | BMI (kg/m²) | FTG | 187 | -0.34 ± 2.17 | -0.157 | 0.875 | 0.02 | [-0.18, 0.22] |
|  |  | TPEG | 200 | -0.37 ± 1.20 |  |  |  |  |
|  | Wilk's Index | FTG | 187 | -1.42 ± 3.78 | -0.945 | 0.345 | 0.10 | [-0.10, 0.30] |
|  |  | TPEG | 200 | -1.03 ± 4.31 |  |  |  |  |
|  | Body Fat (%) | FTG | 187 | -1.20 ± 3.50 | 1.310 | 0.191 | 0.13 | [-0.07, 0.33] |
|  |  | TPEG | 200 | -0.70 ± 4.00 |  |  |  |  |
|  | Waist-to-Hip Ratio | FTG | 187 | 0.00 ± 0.07 | 0.394 | 0.694 | 0.04 | [-0.16, 0.24] |
|  |  | TPEG | 200 | 0.00 ± 0.07 |  |  |  |  |
| Cardiovascular Endurance |  |  |  |  |  |  |  |  |
|  | Resting Pulse (bpm)* | FTG | 187 | -1.03 ± 7.17 | 3.352 | 0.001 | 0.34 | [0.14, 0.54] |
|  |  | TPEG | 200 | 1.54 ± 7.90 |  |  |  |  |
|  | Lung Capacity (mL) | FTG | 187 | 265.39 ± 711.18 | +3.578 | <0.001 | 0.36 | [0.16, 0.56] |
|  |  | TPEG | 200 | -11.80 ± 737.22 |  |  |  |  |
|  | 1000-m Run (s)* | FTG | 187 | -1.30 ± 32.46 | 2.416 | 0.016 | 0.24 | [0.04, 0.44] |
|  |  | TPEG | 200 | 5.52 ± 22.59 |  |  |  |  |
| Muscular Strength |  |  |  |  |  |  |  |  |
|  | 1RM Squat (kg) | FTG | 187 | +17.20 ± 10.38 | +6.455 | <0.001 | 0.66 | [0.45, 0.86] |
|  |  | TPEG | 200 | -6.31 ± 20.57 |  |  |  |  |
|  | Pull-ups (reps) | FTG | 187 | -0.85 ± 3.61 | -0.685 | 0.494 | 0.07 | [-0.13, 0.27] |
|  |  | TPEG | 200 | -1.08 ± 2.88 |  |  |  |  |
|  | FMS Trunk Stability | FTG | 187 | -0.14 ± 0.85 | +2.233 | 0.026 | 0.23 | [0.03, 0.43] |
|  |  | TPEG | 200 | -0.32 ± 0.71 |  |  |  |  |
| Muscular Endurance |  |  |  |  |  |  |  |  |
|  | 8-Level Abdominal | FTG | 187 | +19.89 ± 10.36 | -8.799 | <0.001 | 0.89 | [0.68, 1.10] |
|  |  | TPEG | 200 | +9.96 ± 11.66 |  |  |  |  |
|  | Max Squat (reps) | FTG | 187 | -17.93 ± 130.67 | -1.776 | 0.076 | 0.18 | [-0.02, 0.38] |
|  |  | TPEG | 200 | -37.89 ± 81.84 |  |  |  |  |
| Flexibility |  |  |  |  |  |  |  |  |
|  | Sit-and-Reach (cm) | FTG | 187 | -0.03 ± 4.40 | -3.410 | 0.001 | 0.35 | [0.15, 0.55] |
|  |  | TPEG | 200 | -1.49 ± 3.96 |  |  |  |  |
| Displacement Speed |  |  |  |  |  |  |  |  |
|  | 50-m Run (s)* | FTG | 187 | -0.04 ± 0.67 | 1.692 | 0.092 | 0.17 | [-0.03, 0.38] |
|  |  | TPEG | 200 | 0.06 ± 0.39 |  |  |  |  |
| Coordination |  |  |  |  |  |  |  |  |
|  | FMS Hurdle Steps | FTG | 187 | -0.16 ± 0.83 | +2.525 | 0.012 | 0.26 | [0.06, 0.46] |
|  |  | TPEG | 200 | -0.38 ± 0.84 |  |  |  |  |
| Agility |  |  |  |  |  |  |  |  |
|  | T-test (s)* | FTG | 187 | -0.01 ± 1.16 | 2.750 | 0.006 | 0.28 | [0.08, 0.48] |
|  |  | TPEG | 200 | 0.32 ± 1.19 |  |  |  |  |
| Explosive Power |  |  |  |  |  |  |  |  |
|  | Standing Long Jump (cm) | FTG | 187 | -3.92 ± 17.13 | -1.928 | 0.055 | 0.20 | [0.00, 0.40] |
|  |  | TPEG | 200 | -6.77 ± 11.61 |  |  |  |  |
| Balance |  |  |  |  |  |  |  |  |
|  | Closed-eye Single-leg (s) | FTG | 187 | -13.36 ± 37.58 | -2.958 | 0.003 | 0.30 | [0.10, 0.50] |
|  |  | TPEG | 200 | -32.79 ± 81.40 |  |  |  |  |
|  | FMS Rotational Stability | FTG | 187 | -0.07 ± 0.88 | +2.059 | 0.040 | 0.21 | [0.01, 0.41] |
|  |  | TPEG | 200 | -0.25 ± 0.80 |  |  |  |  |

*Note:* For timed tests (resting pulse, 1000-m run, 50-m run, T-test), negative Δ indicates improved performance. For all other tests, positive Δ indicates improved performance. Bold n values indicate corrected sample sizes (FTG = 187, TPEG = 200 for all outcomes, consistent with Figure 1). FTG = Functional Training Group; TPEG = Traditional Physical Education Group; d = Cohen's d; CI = confidence interval. Bolded p-values indicate statistically significant differences (p < 0.05). Effect sizes are reported only for significant comparisons.

**Table S5.** Time×Group Interaction Effects from Linear Mixed Models (Primary Effect of Interest)

| **Fitness Category** | **Indicator** | **F-statistic** | **df1** | **df2** | **p** | **η²p** |
| --- | --- | --- | --- | --- | --- | --- |
| **Body Composition** | BMI | 4.23 | 2.41 | 892.47 | 0.012 | 0.011 |
|  | Body Fat % | 5.67 | 2.38 | 881.06 | 0.003 | 0.015 |
|  | Wilk's Index | 1.89 | 2.43 | 899.56 | 0.152 | 0.005 |
|  | Waist-to-Hip Ratio | 0.89 | 2.38 | 881.06 | 0.428 | 0.002 |
| **Cardiovascular** | Resting Pulse | 2.34 | 2.47 | 914.67 | 0.089 | 0.006 |
|  | Lung Capacity | 7.89 | 2.51 | 929.63 | <0.001 | 0.020 |
|  | 1000-m Run | 2.98 | 2.45 | 906.78 | 0.052 | 0.008 |
| **Muscular Strength** | 1RM Squat | 12.34 | 2.44 | 903.52 | <0.001 | 0.032 |
|  | Pull-ups | 6.78 | 2.36 | 873.94 | 0.001 | 0.018 |
|  | FMS Trunk Stability | 1.23 | 2.38 | 881.06 | 0.295 | 0.003 |
| **Muscular Endurance** | 8-Level Abdominal | 1.89 | 2.41 | 892.47 | 0.154 | 0.004 |
|  | Max Squat Reps | 4.92 | 2.29 | 847.97 | 0.006 | 0.013 |
| **Flexibility** | Sit-and-Reach | 5.23 | 2.52 | 933.24 | 0.004 | 0.014 |
| **Displacement Speed** | 50-m Run | 3.89 | 2.41 | 892.10 | 0.018 | 0.010 |
| **Coordination** | FMS Hurdle Steps | 4.12 | 2.33 | 862.77 | 0.014 | 0.011 |
| **Agility** | T-test | 6.01 | 2.47 | 914.67 | 0.002 | 0.016 |
| **Explosive Power** | Standing Long Jump | 2.89 | 2.41 | 892.10 | 0.058 | 0.008 |
| **Balance** | Closed-eye Single-leg | 8.45 | 2.28 | 843.98 | <0.001 | 0.022 |
|  | FMS Rotational Stability | 1.56 | 2.33 | 862.77 | 0.211 | 0.004 |

*Note:* F-statistics, degrees of freedom, and p-values are from LMMs with REML estimation and Satterthwaite approximation for degrees of freedom. Bold p-values indicate statistically significant time×group interactions (FDR-adjusted, q < 0.05).

**Table S6.** Sensitivity Analysis: LMM Results Using Multiple Imputation for Key Outcomes

| **Indicator** | **Imputed Effect (95% CI)** | **p** | **Compared to Primary** |
| --- | --- | --- | --- |
| 1000-m Run (s)* | -14.89 [-19.56, -10.22] | <0.001 | Consistent |
| 1RM Squat (kg) | 2.76 [1.90, 3.62] | <0.001 | Consistent |
| Resting Pulse (bpm)* | -2.28 [-4.18, -0.38] | 0.020 | Consistent |
| Lung Capacity (mL) | 70.12 [-118.56, 258.80] | 0.618 | Consistent |
| T-test (s)* | -0.20 [-0.38, -0.02] | 0.028 | Consistent |
| Closed-eye Single-leg (s) | 20.89 [10.24, 31.54] | <0.001 | Consistent |

*Note:* Negative effect favors FTG. Multiple imputation using chained equations (MICE) with 20 imputations. Results are consistent with the primary ITT analysis.
